# On the Effect of Modified Carbohydrates on the Size and Shape of Gold and Silver Nanostructures

**DOI:** 10.3390/nano10071417

**Published:** 2020-07-21

**Authors:** Idris Yazgan, Abdurrahman Gümüş, Kutalmış Gökkuş, Mehmet Ali Demir, Senanur Evecen, Hamide Ayçin Sönmez, Roland M. Miller, Fatma Bakar, Ayhan Oral, Sergei Popov, Muhammet S. Toprak

**Affiliations:** 1 Center of Biosensors and Materials, Department of Biology, Faculty of Science and Arts, Kastamonu University, 37150 Kastamonu, Turkey; 115411013@ogr.kastamonu.edu.tr; 2 Department of Electrical and Electronics Engineering, İzmir Institute of Technology, 35430 İzmir, Turkey; abdurrahmangumus@iyte.edu.tr; 3 Department of Environmental Engineering, Kastamonu University, 37150 Kastamonu, Turkey; kgokkus@kastamonu.edu.tr; 4 Center of Science and Art, 37150 Kastamonu, Turkey; senanurevecen@gmail.com (S.E.); sonmezaycn55@gmail.com (H.A.S.); fbakar37@hotmail.com (F.B.); 5 Department of Chemistry, SUNY Binghamton University, Binghamton, NY 13902, USA; rmiller8@binghamton.edu; 6 Department of Chemistry, Çanakkale Onsekiz Mart University, 17100 Çanakkale, Turkey; ayhanoral@comu.edu.tr; 7 Department of Applied Physics, KTH Royal Institute of Technology, SE106 91 Stockholm, Sweden; sergeip@kth.se

**Keywords:** sugar ligands, modified carbohydrates, colloidal synthesis, plasmonics, gold-Au, silver-Ag, nanoparticles, morphology control

## Abstract

Gold (Au) and silver (Ag) nanostructures have widespread utilization from biomedicine to materials science. Therefore, their synthesis with control of their morphology and surface chemistry have been among the hot topics over the last decades. Here, we introduce a new approach relying on sugar derivatives that work as reducing, stabilizing, and capping agents in the synthesis of Au and Ag nanostructures. These sugar derivatives are utilized alone and as mixture, resulting in spherical, spheroid, trigonal, polygonic, and star-like morphologies. The synthesis approach was further tested in the presence of acetate and dimethylamine as size- and shape-directing agents. With the use of transmission electron microscopy (TEM), selected area electron diffraction (SAED), x-ray diffraction (XRD), scanning electron microscopy (SEM), and ultraviolet-visible (UV-vis) absorption spectroscopy techniques, the particle size, shape, assembly, aggregation, and film formation characteristics were evaluated. NPs’ attributes were shown to be tunable by manipulating the sugar ligand selection and sugar ligand/metal-ion ratio. For instance, with an imine side group and changing the sugar moiety from cellobiose to lactose, the morphology of the Ag nanoparticles (NPs) transformed from well dispersed cubic to rough and aggregated. The introduction of acetate and dimethylamine further extended the growth pattern and morphological properties of these NPs. As examples, L5 AS, G5AS, and S5AS ligands formed spherical or sheet-like structures when used alone, which upon the use of these additives transformed into larger multicore and rough NPs, revealing their significant effect on the NP morphology. Selected samples were tested for their stability against protein corona formation and ionic strength, where a high chemical stability and resistance to protein coating were observed. The findings show a promising, benign approach for the synthesis of shape- and size-directed Au and Ag nanostructures, along with a selection of the chemistry of carbohydrate-derivatives that can open new windows for their applications.

## 1. Introduction

Gold (Au) and silver (Ag) nanomaterials have applications in various fields including biomedicine, energy storage, drug development and delivery, sensor technologies, environmental remediation, and electronic displays [1,2,3,4]. In particular, wet-chemistry-mediated synthesis of these nanostructures are of great interest owing to their flexible design in controlling the size, shape, and surface chemistry [5]. However, classical approaches require multiple steps including synthesis, activation, isolation, and functionalization. These steps cause increased byproduct formation, toxic chemical utilization while limiting large scale, inexpensive, and benign production accompanied by high reproducibility [6,7]. Therefore, methods free of toxic byproducts with minimal process steps are under intensive investigation.

Biomolecules including amino acids, peptides [8,9], sugars, and sugar polymers [10] were shown to act as reducing, capping, and stabilizing agents in the synthesis of metallic nanostructures including Au and Ag [11,12,13]. However, utilization of plain sugars and amino acids for synthesis do not provide precise control of morphology, size, and dimensions of Au and Ag nanostructures with long-term stability. Therefore, the introduction of size- and shape-directing agents along with extra stabilizing agents is required for the precise control of the synthesis process [6].

Click chemistry, ligand exchange, electrostatic, and molecular interactions are the common approaches to engineer the surface chemistry of metallic nanomaterials [3]. Surface chemistry, along with the shape and size, dictates the optical properties of nanostructures [14]. In addition, surface chemistry is the most dominant parameter determining the self-aggregation behavior [15]. For example, morphological anisotropy brings novel capabilities to both Au and Ag nanoparticles (NPs) [16]. Seed-mediated growth, electrochemistry, and sonochemistry are among the most common techniques in the synthesis of anisotropic metallic NPs [16].

Colloidal NPs behave as building blocks to develop higher-order structures including 1D and 3D structures and films [10,17], which may bring along unprecedented electronic and optical properties such as enhanced surface plasmon resonance (eSPR), owing to gathering of oscillating electric fields of adjacent NPs [15,18,19]. Charge–charge interactions resulting from the surface chemistry of plasmonic NPs and proper surfactant utilization may trigger self-assembly of Au-NPs [8] and Ag-NPs [20]. NP-based films can be synthesized through different mechanisms, including a spontaneous process dictated by the surface chemistry and the environment, or by an external force relying on complicated and fine procedures [20].

In this study, we developed derivatives of simple carbohydrates (sugar derivatives or sugar ligands) via substitution of organic molecules and used them to synthesize shape- and size-controlled Au and Ag nanostructures, where no additional reducing, shape-directing, and/or stabilizing agents were needed. Spherical, non-spherical, star-shaped, polygonic, and sheet-like Au and Ag nanostructures were obtained depending on the chemistry of the sugar ligand and sugar ligand/metal salt ratio. Further alteration in size and shape along with self-assembly was observed in the presence of dimethyl amine and acetate. The findings show that by utilizing sugar derivatives, highly stable colloidal Au and Ag nanostructures with different morphologies can be easily synthesized at room temperature.

## 2. Materials and Methods

### 2.1. Materials

D-Mannose (C_6_H_12_O_6_; ≥99%), D-Galactose (C_6_H_12_O_6_; ≥99%), D-Sucrose (C_12_H_22_O_11_; ≥99.5%), D-Lactose monohydrate (C_12_H_22_O_11_·H_2_O; ≥99.5%), D-Cellobiose (C_12_H_22_O_11_; ≥99%), *p*-aminobenzoic acid (H_2_NC_6_H_4_CO_2_H; ≥99%), 4-aminophenyl ether (O(C_6_H_4_NH_2_)_2_; ≥97%), 4,4′-diaminobibenzyl (C_14_H_16_N_2_; ≥95%), 5-aminosalicylic acid (H_2_NC_6_H_3_-2-(OH)CO_2_H; ≥99%), 3-aminophenol (H_2_NC_6_H_4_OH; ≥98%), Penicillamine ((CH_3_)_2_C(SH)CH(NH_2_)CO_2_H; 99%), silver nitrate (AgNO_3_; ≥99%), and Gold (III) chloride hydrate (HAuCl_4_·xH_2_O; ≥99.995%) were purchased from Sigma–Aldrich (Ankara, Turkey). Glacial acetic acid, anhydrous acetone, anhydrous ethanol, anhydrous methanol and sodium chloride, and potassium perchlorate were purchased from Merck (Ankara, Turkey). All the chemicals were used as they were received from the manufacturer, without further purification. Deionized water (DI) (18.2 MΩ) was produced using a Zeneer purification system (Seoul, Korea).

### 2.2. Synthesis, Purification, and Characterization of Sugar Ligands

**Figure 1:**
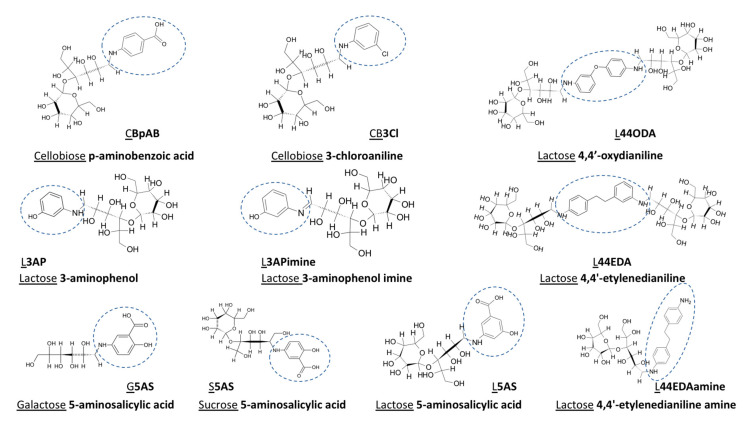
Molecular structure and names of the synthesized sugar ligands. Cellobiose **p-aminobenzoic acid** (CB**pAB**); Cellobiose **3-chloroaniline** (CB**3Cl**); Lactose **3-aminophenol** (L**3AP**); Lactose **3-aminophenol imine** (L3**APimine**); Lactose **4,4′-oxydianiline** (L**44ODA**); Lactose **4,4′-ethylenedianiline** (L**44EDA**); Lactose **4,4′-ethylenedianiline amine** (L**44EDAamine**); Galactose **5-aminosalicylic acid** (G**5AS**); Sucrose **5-aminosalicylic acid** (S**5AS**); Lactose **5-aminosalicylic acid** (L**5AS**). Functional groups on the sugar ligands are indicated with a dashed circle in the figure, and their abbreviations are indicated with bold characters (sugar groups and their abbreviations are underlined in the sketch only). Theoretical properties are given in Supplementary Information, Table S1.

Sugar ligands were synthesized via two-step reductive amination methods as described in detail in earlier reports [21,22]. Thin layer chromatography (TLC) (Merck, Ankara) in hydrophilic interaction chromatography mode was used to monitor completion of the reaction while flash chromatography equipped with a C18 column was preferred for further purification of the products whenever needed. The structure of the synthesized sugar ligands along with their full and abbreviated names is presented in Figure 1. (The functional group on each sugar ligand is indicated by a dashed circle to make it easier to follow.)

### 2.3. Synthesis of Gold and Silver Nanoparticles

The synthesis procedure was a one-step procedure. Briefly, stock solutions of sugar ligands, AgNO_3_ (100 mg/mL), and HAuCl_4_·xH_2_O (25 mg/mL) were prepared in DI water. Different ratios of sugar ligands/metallic salts were mixed to obtain Au-NPs and Ag-NPs, where no additional capping and/or stabilizing agents were used. All the reactions were completed within 30 min except those forming larger particles; these are explicitly stated under the relevant figures. Details of the prepared samples using the synthesized sugar ligands are listed in Table 1. Sample names in Table 1 are expressed as a combination of the sugar ligand abbreviation, followed by the material synthesized and the molar ratio of the sugar ligand to metal ions in the given sequence. When dimethyl amine and acetate were included, suffixes *y* and *z* were added to define the dimethylamine: metal salt and acetate: metal salt mole ratios, respectively.

### 2.4. Characterization Methods

UV-vis analysis of pure sugar ligands and Au and Ag NPs was performed on a PG Instruments T60 Visible Spectrophotometer (Leicestershire, UK), within a 190–1100 nm wavelength range using quartz cuvettes (range 190–2500 nm). Surface plasmon (SP) absorption (also referred to as surface plasmon resonance–SPR) of each Au NP and Ag NP colloid was measured; dilutions of the NP colloids were performed using DI water to reach absorbance values below 1.5.

Morphological characterization of NPs was performed using transmission electron microscopy (TEM, Hitachi at 220 kV, Tokyo, Japan; HR-TEM, FEI TALOS F200S TEM 200 kV, Hillsboro, OR, USA), and Scanning Transmission Electron Microscopy (FEI QUANTA 250 FEG, Oregon, USA). Standard 300-mesh (01895-F, Ted-Pella, CA, USA) and 400-mesh (01896N, Ted-Pella, CA, USA) TEM grids were used for the analyses; samples were prepared by drop casting and drying 200 µL of respective colloids on the grid. Morphological characterization of large nanostructures was performed using OMAX 40X-2500X LED Digital Trinocular light-microscopy (amazon.com, USA). X-Ray powder diffraction (XRPD) of Au and Ag NPs was performed using a Bruker D8 Advance Diffractometer (MA, USA). NP colloids were precipitated by centrifugation, followed by freezing at −82 °C. Frozen samples were then lyophilized overnight using a Christ Alpha 1–2 LD Freeze Dryer lyophilizer (Osterode am Harz, Germany).

Stability evaluation of the selected Au and Ag NPs was performed in the presence of up to 1.0 M NaCl, up to 7.0 M L-penicillamine, 1.0 M potassium perchlorate and freshly prepared nutrient broth. All the tests, except protein corona formation, were done at room temperature. Protein corona tests were performed at 37 °C for 2 h. Lyophilized Au and Ag NPs (whose SPR peaks were below 1.0 absorbance unit (a.u) were introduced to the test media. Shifts of SPR peaks monitored by UV-vis spectroscopy were used as an indicator of vulnerability of the Au and Ag NPs against the tested conditions.

## 3. Results and Discussion

Synthesis of Au and Ag NPs from their ions requires redox chemistry, where the electrons are provided simply by the reducing agents, hereby represented by the sugar ligands. Various functionalities have been used for various sugar ligands. Cellobiose (CB) is derivatized by aminobenzoic (pAB) acid and 3-chloroaniline (3Cl) ligands, while lactose (L) through aminophenol (3AP), aminophenol-imine (3APimine), 4,4-oxydianiline (44ODA), 4,4-ethylenedianiline (44EDA), and 4,4-ethylenedianiline+amine (44EDAamine); lastly 5-amino salicylic acid (5AS) acid functionalization was performed on three different sugar ligands: galactose (G), L, and sucrose (S). The effect of sugar ligands on the formation, morphology, and size of Au and Ag nanostructures is detailed in the sections below. Our findings of this rich carbohydrate chemistry are summarized in a table towards the end of the manuscript.

### 3.1. Au NPs Synthesized Using Cellobiose Sugar Ligands Derivatized with P-Aminobenzoic Acid (CBpAB) 

**Figure 2:**
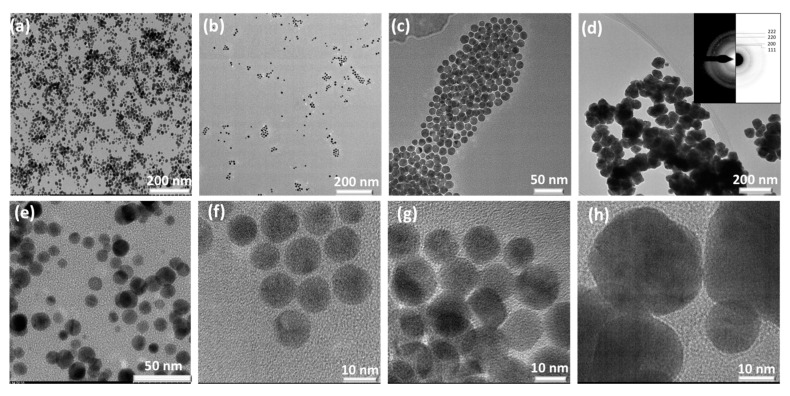
Characterization of some Au NPs synthesized using Cellobiose **p-aminobenzoic acid** (CB**pAB**)-derivatized sugar ligands. TEM micrographs of (**a**,**e**) CB**pAB**-Au_38; (**b**,**f**) CB**pAB**-Au_26; (**c**,**g**) CB**pAB**-Au_13; (**d**,**h**) CB**pAB**-Au_5. Inset in (d) is a representative SAED pattern of Au NPs synthesized using CB**pAB**. SAED pattern in (**d**) is indexed for the cubic (fcc) lattice of Au (ICDD PDF: 00–004–0784). (For molecular structures and sample designations see Figure 1 and Table 1).

**Figure 3:**
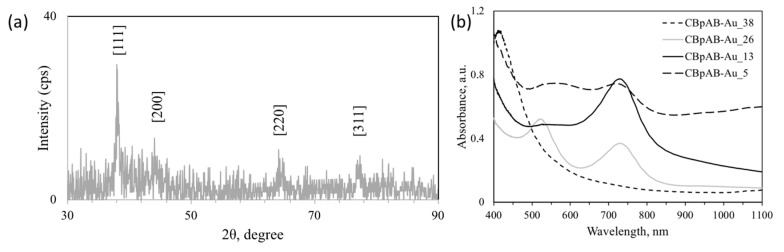
(**a**) Typical powder X-ray diffraction (PXRD) pattern (indexed for the cubic (fcc) lattice of Au (ICDD PDF: 00-004-0784)) and (**b**) UV-vis absorption spectra of samples presented in Figure 2. (For sample designations see Table 1).

Several samples were synthesized by varying the ratio of CBpAB to Au^3+^ (Figure 2) to study the effect of this ratio on the NPs formed. Detailed sample designations are presented in Table 1. The formation of all CBpAB-Au NPs took place within 30 min. The first attempt was made with CBpAB-Au_38. Spherical Au particles with an average diameter of 10 nm were obtained (Figure 2a,e). The sample had a distinctive plasmon absorption at 530 nm as presented in Figure 3b. Decreasing the ratio of CBpAB/Au^3+^ triggered self-assembly of Au NPs without an accompanied increase in polydispersity of the particles. When the CBpAB/Au^3+^ ratio was decreased to 5, dramatic changes were observed in the morphology and size of Au NPs (Figure 2d,h). UV-vis spectra presented in Figure 3b reveal two absorption peaks at~540 and 740 nm with a decreasing CBpAB/Au^3+^ ratio. UV-Vis analysis of pure CBpAB did not reveal any clear absorption features (Supplementary information, Figure S1). The absorption of Au colloids at longer wavelengths can be ascribed to the agglomeration of small Au NPs with a decreasing CBpAB/Au^3+^ ratio where charge transfer between the NPs can be facilitated by charge-bearing, surface-bound sugar ligands. Utilization of different sugar ligands including cellobiose 4,4′-Diaminodiphenyl sulfone at a low sugar ligand/Au^3+^ ratio resulted in agglomerated Au NPs with more than one SPR peak (Supplementary information, Figure S2).

Synthesized NPs were crystalline as can be seen from the SAED pattern showing diffraction spots/rings corresponding to (111), (200), (220), and (311) crystal planes of fcc Au in Figure 2d (ICDD PDF: 000-004-0784; International Centre for Diffraction Data). The crystallinity of synthesized Au NPs was also investigated by PXRD, and the corresponding diffraction pattern is shown in Figure 3a. Au nanocrystals exhibited four distinct diffraction peaks at 2θ = 38.1° 44.3°, 64.5°, and 77.7°. All observed peaks correspond to the face center cubic (fcc) lattice of Au, where Bragg reflections (111), (200), (220), and (311) are indexed on the pattern, in agreement with the SAED results.

Using CBpAB for Ag synthesis did not yield any NP formation within 6 h, where the solution color only barely changed after 16 h. Therefore, further synthesis attempts with Ag were not performed. The difference in the NP formation time can be explained by the thermodynamics of the process, based on the reduction potential of these two elements. Gold has a reduction potential of +1.42 V, which allows formation of NPs even with moderate strength reducing agents, while Ag^+^ (+0.80 V), with a lower reduction potential, may not be reduced as easily/favorably.

### 3.2. Au NPs Synthesized Using Cellobiose Sugar Ligands Derivatized with 3-Chloroaniline (CB3Cl) 

**Figure 4:**
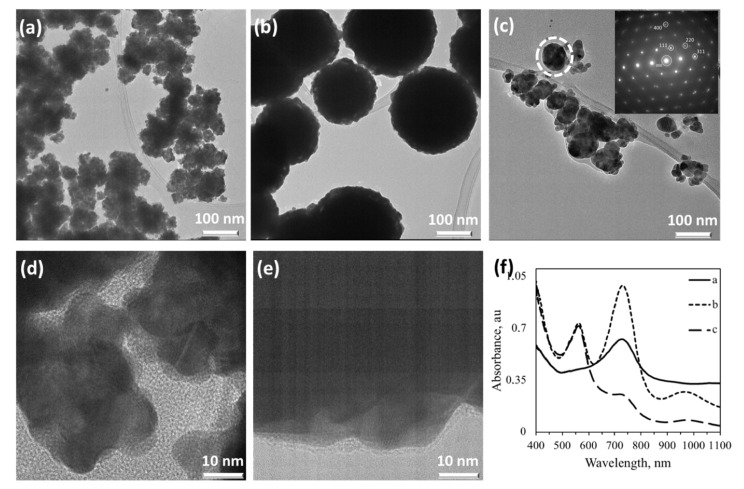
Characterization of some Au NPs synthesized using Cellobiose **3-chloroaniline** (CB**3Cl**) derivatized sugar ligands. TEM micrographs of (**a**,**d**) CB**3Cl**-Au_53; (**b**,**e**) CB**3Cl**-Au_8a isolated and (**c**) CB**3Cl**-Au_8b (inset: SAED of circled particle), and (**f**) UV-vis absorption spectra of samples (**a**,**c**). (For molecular structures and sample designations see Figure 1 and Table 1).

CB3Cl-synthesized Au NPs showed a similar dependency of particle size on the sugar ligand/Au^3+^ ratio (Figure 4). At a high ratio (53), NPs formed more aggregates while at a lower ratio (8) more spherical NPs were obtained (Figure 4a,c). The NPs shown in Figure 4b,c were from the same batch; the ones in Figure 4b were formed 30 sec after sugar ligand-Au salt mixing (by pipetting 100 µL of the sample into 900 µL of 18.2 MΩ DI water). It is highly possible that the NP formation did not come to completion within 30 sec, and surface chemistry played the key role in the formation of larger particles; 30 min was therefore chosen as the standard incubation time for full formation of Au NPs with this synthetic method. The SAED pattern given in the inset of Figure 4c reveals that larger NPs forming upon prolonged reaction duration are single crystalline. Particle agglomeration can easily be monitored from the UV-vis spectra presented in Figure 4f, where a vague plasmon absorption typical of Au NPs is observed for the CB3Cl-Au_53 sample at 520 nm with a rather strong absorption centered around 750 nm. The absorption at 750 nm corresponds to an Au NP size of about 150 nm; particles in this range are also visible from Figure 4a. The CB3Cl-Au_8 sample also shows two absorptions at longer wavelengths, centered around 750 nm and 1000 nm, besides the one at 520 nm. When the particle size exceeded 200 nm, an increase in plasmon absorptions can be seen for Au nanostructures. Therefore, although the smaller ratio leads to more spherical particles, it also leads to particle reformation along with the formation of larger polycrystalline particles.

The thermodynamics of Ag NP formation using CB3Cl were similar to that observed for CBpAB, where no NP formation was observed upon prolonged reaction periods. Therefore, no further synthesis attempts of Ag NPs were performed using CB3Cl.

A comparison of CBpAB and CB3Cl shows that spherical and well-separated Au NPs in the order of 5–10 nm could be prepared by using CBpAB, while CB3Cl yielded more aggregated and bulky nanostructures. The side group of p-aminobenzoic acid, therefore, was shown to be more effective in morphology and size control of Au NPs. Neither of the sugar ligands was found to be active for Ag^+^ reduction.

### 3.3. Ag NPs synthesized Using Lactose Sugar Ligands Derivatized with Aniline (L44EDA, L44ODA), Amine (L3AP), Imine (L3APimine, L44EDAimine) Groups 

**Figure 5:**
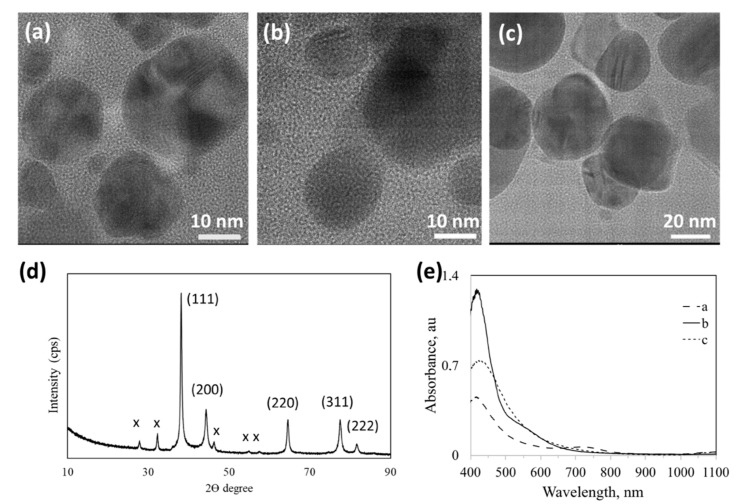
Characterization of Ag NPs synthesized using various **aniline-**bearing sugar ligands. (**a**) L**44ODA**-Ag, (**b**) L**44EDA**-Ag_0.5 and (**c**) L**44EDA**-Ag_2; (**d**) typical X-ray powder diffraction pattern of Ag NPs indexed for fcc Ag (ICDD PDF: 00-004-0783; x marked peaks are attributed to crystalline carbohydrate residues in the dried sample for XRD analysis), and (**e**) UV-vis absorption spectra for samples in a–c. (For molecular structure of ligands and sample designations see Figure 1 and Table 1).

Aniline containing L44ODA and L44EDA sugar ligands has been used for the synthesis of Ag NPs, which showed much more favorable thermodynamics and faster reduction kinetics for Ag. Figure 5 reveals that a lower sugar-ligand/Ag^+^ ratio produced spherical particles in comparison to the synthesis of sugar ligand-mediated Au NPs. An increase in the L44EDA/Ag^+^ ratio did not cause dramatic changes in NPs’ size and shape (Figure 5b,c) while it strongly affected the crystallinity of the formed Ag NPs. UV-vis spectra of the three Ag NP samples with amine-derivatized sugar ligands are presented in Figure 5e, where a strong plasmon absorption is observed at 430 nm, typical for Ag NPs. The position of absorption maximum reveals a similar size of the particles formed. The width of the absorption profiles reveals the size dispersity, where a wider peak refers to a wider size distribution in a particular NP solution. The imine form of the ligands shows absorption between 480 and 550 nm (L44EDAimine, Figure S1), which is related to delocalization of π electrons through –C–C– of the benzene ring and C=N of the imine group. This is usually visible as a weak shoulder in the spectra displayed in Figure 5e. the L44EDA-Ag_2 sample shows the broadest size distribution, while the L44ODA-Ag sample shows a weak absorption around 800 nm and L44EDA-Ag_0.5 at 630 nm ascribed to agglomeration/clustering of NPs reaching a size of about 150 nm. The X-ray diffraction pattern of typical L44EDA-Ag (Figure 5d) reveals that Ag NPs possess an fcc crystal structure. The non-assigned peaks (marked with x) were attributed to crystalline organic phases including sugar ligands, in agreement with the literature [23]. L44ODA (and L44EDA) ligand also proved very successful in the synthesis of spherical Au NPs, while changing the L44ODA/Au^3+^ ratio did not alter the size and shape of the NPs formed (results from another dedicated study are under review elsewhere [24]).

**Figure 6:**
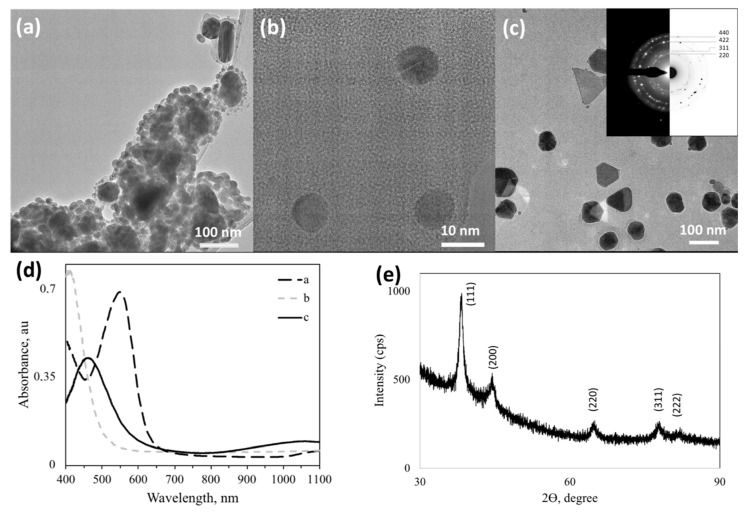
Characterization of Ag NPs synthesized using various **imine-** and **amine-derivatized** sugar ligands. TEM images of (**a**) L3**APimine**-Ag, (**b**) L**3AP**-Ag; (**c**) L**44EDA**:L**44EDAimine**-Ag; (**d**) UV-Vis spectra of samples in (**a**,**c**), and (**e**) Representative XRPD pattern of L**3APimine**-Ag presented in (**a**) indexed for fcc Ag (ICDD PDF: 00–004–0783). (For molecular structure of ligands and sample designations see Figure 1 and Table 1).

In order to investigate the effect of various functional groups on the formation of Ag NPs, amine and imine-derivatized ligands were used during the synthesis; namely, L3AP, L3APimine L44EDA and L44EDAimine (see Figure 1 for structural details). Figure 6 reveals that the imine (Figure 6a) and amine form (Figure 6b) of the same sugar ligand at the same sugar ligand/Ag^+^ ratio resulted in the formation of differently shaped Ag NPs. The amine form of L3AP yielded perfectly spherical and homogenous Ag NPs (~10 nm sized, Figure 6b) while the L3APimine form yielded heterogenous Ag NPs with a large core surrounded by small satellite particles, of which most were between 15–20 nm while some anisotropic NPs were observed with a size ranging between 70 and 100 nm (Figure 6a). In the case of utilizing sugar ligands as a shape-directing agent, mixing L44EDA with a free amino group containing L44EDA (L44EDA:L44EDAamine-Ag) resulted in the formation of anisotropic Ag NPs with mixed morphology of truncated polygons, trigons, and spheroids (Figure 6c). In contrast to Ag, Au NPs did not provide stable nanostructures in the presence of the L44EDA amine. The SAED of this sample, presented in the inset of Figure 6c, revealed the polycrystalline character of these NPs, and diffractions rings indexed to fcc Ag (ICDD PDF: 65-2871) have been marked with the corresponding Miller indices. UV-vis absorption spectra of these samples are presented in Figure 6d, where significant differences are observed. The sample prepared using L3APimine (Figure 6a) exhibited a solution color of pink, with plasmon absorption at 550 nm. The uniformly sized Ag NPs (10 nm) prepared using L3AP (Figure 6b) yielded an SPR peak at 410 nm. Ag NPs with mixed morphology in Figure 6c, exhibiting yellowish color, showed an SPR absorption at 460 nm accompanied by absorption in the near-infrared region due to the co-presence of large and small Ag NPs as well as anisotropy. If spherical and well-dispersed NPs of Ag are desired, for instance, then the ideal choice among the amine-imine ligands would be L3AP.

### 3.4. Ag NPs Synthesized Using Cellobiose Imine and Galactose Aminosalicylic Acid Sugar Ligands 

**Figure 7:**
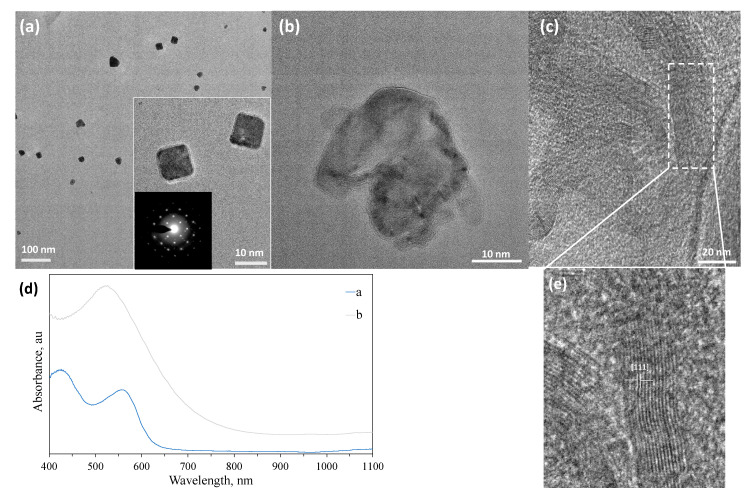
Characterization of single crystalline Ag NPs. (**a**) CB3APimine-Ag, (**b**,**c**) G5AS-Ag, and (**d**) UV-vis spectra of -a and -b. (**e**) Zoomed-in section marked with a rectangle in (**c**) (For molecular structures and sample designations see Figure 1 and Table 1).

In a few cases, nearly single crystalline NPs were obtained for Ag. Introduction of 3AP to cellobiose 3-aminophenolimine (CB3APimine) in the synthesis of Ag NPs resulted in NPs with cubic morphology with an average diameter of ~100 nm (Figure 7a). These NPs showed a single crystalline character as assessed by their SAED pattern (inset of Figure 7a), where the stabilized surface seems to be (100), which is a populated higher energy surface. Here, the dominant effect is ascribed to CB part, as the L ligand with the same functionality generated spherical core-satellite type of morphology (see Figure 6a) Increasing the CB3APimine/Ag ratio up to two times eliminated the formation of single crystalline NPs (data not shown). Micrographs of the G5AS sample, Figure 7b,c, show highly ordered sheets/planes where the measured distance corresponds to the (111) plane of Ag. G5AS seems to stabilize the (111) plane in this case, which is the lowest surface energy plane (0.76 J/m^2^) in the crystal structure of Au, causing anisotropic growth of Ag NPs. The aldehyde group in G may have caused the preferential adsorption on this surface, thus stabilizing it. The CB3APimine-Ag NP sample displays a strong plasmon absorption centered around 540 nm, while G5AS-Ag displays two absorption bands centered at 430 nm and 560 nm. This may be due to the elongated morphology (Figure 7e) of crystalline domains. All of these absorption wavelengths are much longer than typical Ag NP absorption, expected to be around 290 nm.

This shows that there is a higher ordering within these materials influencing the plasmon absorption characteristics, which is dominated by the sugar ligands used. It is reasonable to assume that the sugar ligands may also enhance charge interactions between the different NPs as they carry a charge in a dispersed state due to the side groups on them, thus causing enhanced absorption at longer wavelengths.

The synthetic process is versatile and can be performed easily for direct particle growth on substrates. To demonstrate that, we attempted to fabricate NP films on both plastic and glass surfaces by following the drop-cast technique (Supplementary information, Figure S5). A very high coverage was obtained on both surfaces, while shape and size optimization did not work as it did for colloidal Ag NP synthesis, which is ascribed to local concentration variations influencing the process of NP formation.

### 3.5. Au NPs Synthesized Using Dimethyl Amine and Acetate Added Salicylic Acid (5AS)-Derivatized Sugar Ligands

**Figure 8:**
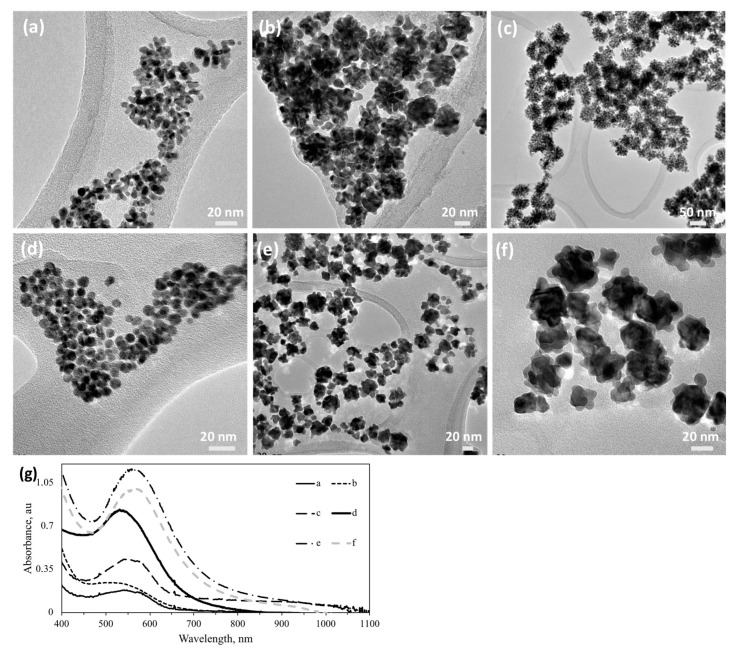
Effect of dimethyl amine and acetate on Au NP synthesis using different **5-amino salicylic acid** (5AS)-derivatized sugar ligands such as: (**a**) S**5AS**-Au_102***yz***; (**b**) S**5AS**-Au_62***yz***; (**c**) S**5AS**-Au_41***yz***; (**d**) G**5AS**-Au_62; (**e**) L**5AS**-Au_62***yz***; and (**f**) G**5AS**-Au_62***yz*** (***y***: dimethylamine: metal salt mole ratio; ***z***: acetate: metal salt mole ratio). (**g**) UV-vis spectra of colloids in (**a**–**f**). (For the molecular structure of sugar ligands and sample designations see Figure 1 and Table 1).

It is well known that molecules with chemically active sites can adsorb onto different crystalline planes and thus influence the resultant NP morphology. We selected dimethyl amine and acetate as the active molecules and studied the effect of their presence on Au NP synthesis, by introducing them into the reaction media. Acetate and dimethyl amine were chosen to evaluate if lone-pair electron-containing species can alter size and shape of the Au NPs. As seen from Figure 8a, spherical Au NPs with a dendritic surface can be synthesized with the S5AS sugar ligand, using acetate and dimethyl amine as size- and shape-directing molecules. However, a decrease in the S5AS/Au ratio yielded self-aggregated Au NPs generally possessing dendrites, i.e., branches (Figure 8b,c) or corona. In the absence of dimethyl amine and acetate, 5-aminosalicylic acid (5AS) containing sugar ligands yielded NPs with spherical morphology as shown in Figure 8d (and Supplementary information, Figure S2). G5AS-mediated synthesis of Au NPs yielded spherical NPs in the range of 8–10 nm (Figure 8d) while at a 4:1 dimethyl amine: acetate ratio, it yielded 15–25 nm star-shaped NPs (Figure 8e). Similar truncated Au NPs can be obtained using LpAB/Au in the presence of dimethyl amine and acetate (Supplementary information, Figure S4). A comparison of Au NPs synthesized using G5AS in the absence (Figure 8d) and presence of dimethyl amine and acetate (Figure 8f) shows a difference in the SP absorption increasing from 540 nm to 560 nm, revealing a smaller average particle in the absence of extra additives. NPs obtained using G5AS (shown in Figure 8f) were similar to L5AS-AuNPs in Figure 8e. The difference between S5AS and L5AS/G5AS could be related to the difference between S and L/G, where the former has a ketone group while the latter possesses an aldehyde group. Au NPs synthesized using only G5AS showed a plasmon absorption at 520 nm (Figure 8g), typical for spherical NPs. Au NPs samples that were made in the presence of acetate and dimethyl amine, independent of sugar ligands, yielded similar morphology which was determined by the SPR peaks centered around 540 nm, revealing no significant NP agglomeration.

**Figure 9:**
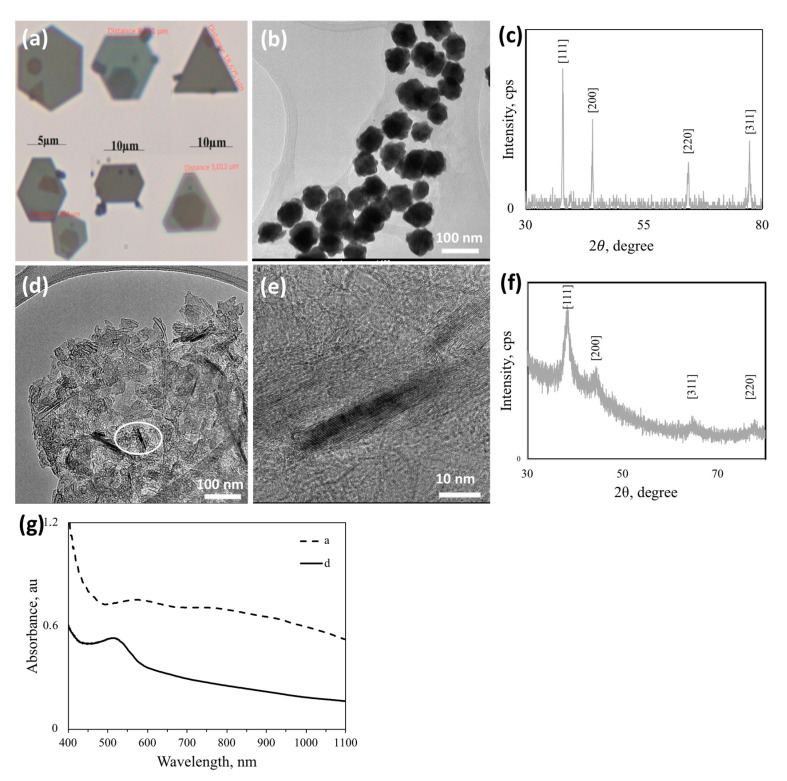
Effect of dimethyl amine and acetate on Au NP synthesis using lactose derivatives; (**a**) L44EDA-Au_0.6 optical micrograph of larger particles (reaction completed within 48 h) (**b**) TEM micrograph of smaller particles in the medium of L44EDA-Au_0.6***y***; (**d**,**e**) TEM micrographs of L5AS-Au_7***yz*** (reaction completed within 8 h) (***y***: dimethylamine: metal salt mole ratio; ***z***: acetate: metal salt mole ratio); (**c**,**f**) PXRD of (**a**) and (**d**) (indexed for the cubic (fcc) lattice of Au (ICDD PDF: 00-004-0784); (**g**) UV-vis spectra of -a and -d. (For sample designations see Table 1 and Figure 1.).

A selected sugar ligand, L44EDA, was used as the reducing agent where size and shape control of the formed Au NPs were overwhelmingly driven by acetate and dimethyl amine. Figure 9a reveals that micron-sized plates with very clear polygon morphologies can be produced within one week of incubation with dimethylamine, while some of the Au NPs from the medium yielded ~60 nm-sized nanostar-like polycrystalline Au NPs (Figure 9b). The PXRD pattern presented in Figure 9c is for the giant hexagonal Au particles in Figure 9a, indexed for the fcc Au (ICDD PDF: 00–004–0784) structure as indicated by the relevant Miller indices. Similarly, the presence of a high amount of dimethyl amine and acetate triggered the formation of Au nanosheets (Figure 9d,e), which are also crystalline as can be observed from the PXRD pattern in Figure 9f. Au NPs in Figure 8e formed at a very high sugar content (L5AS-Au_62*yz*), while those in Figure 9d at a lower sugar and higher acetate-dimethylamine content (L5AS-Au_7*yz*). PXRD in Figure 9c is more representative of isotropic NPs while that in Figure 9f shows a preferential growth direction of (111), which seems to be a synergistic effect of the co-presence of certain dimethyl amine- and acetate-stabilizing planes and enhanced anisotropic, directional growth. Figure 9g shows that larger AuNPs (Figure 9a) yielded a very broad SPR spectrum from 520 nm to 1100 nm, with clear peak centers at ~590 nm and 805 nm. In contrast to this, the nanosheet Au sample (Figure 9d) yielded a narrower SPR spectrum with a peak center at ~530 nm.

A summary of NP morphologies, their size, and size distribution are summarized in Table 2. Depending on the morphology and size of interest, this table can help to choose the proper sugar ligand for Au and Ag NP synthesis. The sugar site is the dominant redox active site, where the derivatizations added on clearly influences the extent of NP aggregation. CB could reduce Au ions with any side group; however, Ag ions were reduced only in the presence of imine derivatization (CB3APimine). This helps to infer that side groups may also participate in the redox process. This kind of selective chemistry/reduction can even be used to separate a mixture of Au and Ag ions by using CBpAB or CB3Cl. Keeping the imine group the same and changing the sugar ligand from CB to L, the morphology of the formed Ag NPs changed from well dispersed cubic (CB3APimine) to rough and aggregated (L3APimine). Here, one can see the effect of the respective sugar group on the morphology of NPs formed. An overview of side group chemistry can be understood by the effect of the investigated lactose (L) ligands on the formation of Ag NPs; L3AP and L44EDA yielded smooth spherical Ag NPs, while L44ODA yielded smooth spherical aggregated NPs, and L3APimine, rough aggregated (>100 nm) NPs. A mixture of amine and imine (L44EDA + L44EDAamine) sugar ligands resulted in anisotropic particles with mixed morphology and a wide range of sizes. Amino salicylic acid functionalization (L5AS) produced sheet-like Ag nanostructures. When extra additives such as dimethyl amine and acetate were utilized for 5AS sugar ligands (L5AS, G5AS, and S5AS), spherical or sheet-like structures transformed into multicore and rough NPs, revealing the significant effect of these additives with lone pairs on the NPs’ morphology.

**Figure 10:**
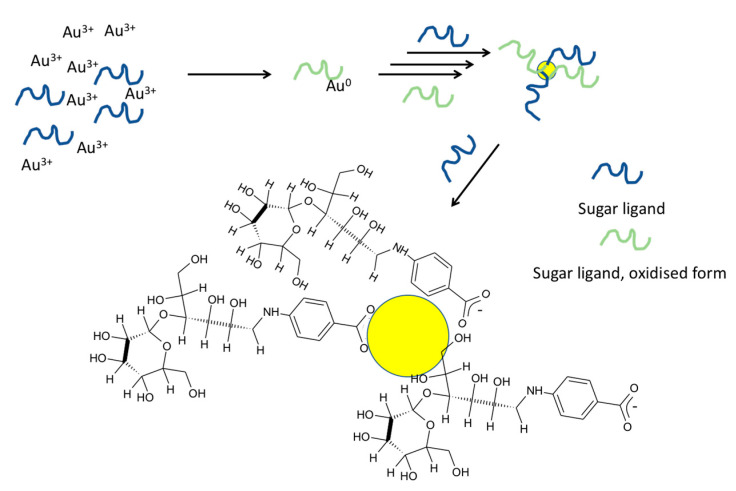
Proposed mechanism for sugar ligand-mediated Au/Ag NPs synthesis. (Resultant oxidized sugar ligand species are not represented in their molecular form as they were not identified/characterized.).

**Figure 11:**
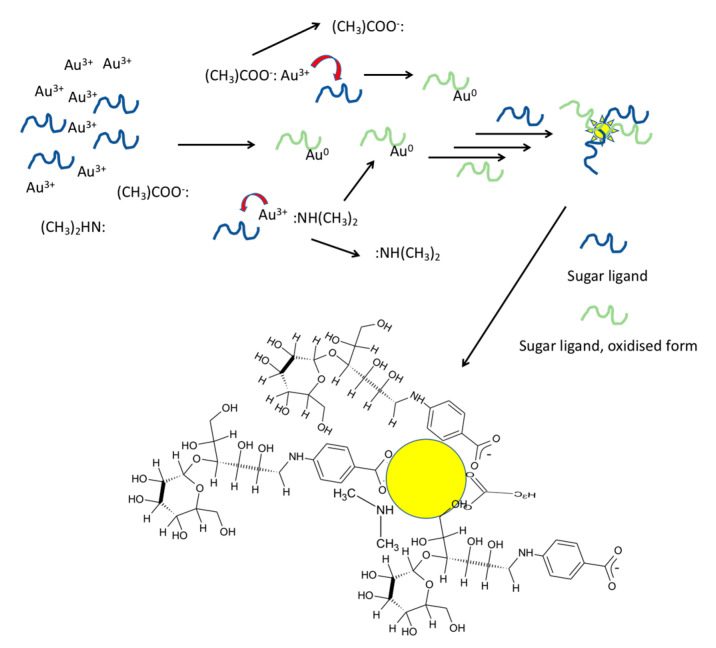
Proposed mechanism for sugar ligand-mediated Au/Ag NPs synthesis in the presence of acetate and dimethylamine. (Resultant oxidized sugar ligand species are not represented in their molecular form as they were not identified/characterized.).

We propose the following mechanism to explain Au/Ag NP formation with the sugar ligands by considering the interaction and molar ratio between the species used. Since sugar ligands were the only agents that reduced Au^3+^/Ag^+^ and provided stability for the formed NPs, there must be a strong interaction between the ligand and formed Au/Ag NPs. According to ^1^H NMR studies, no new shifts of the sugar ligands occurred upon NP formation except L44EDA_0.6, under a very low concentration of the sugar ligand in comparison to Au^3+^ (Supplementary information, Figures S6 and S7). This could be because only a small fraction of the sugar ligand was oxidized in response to NP formation, so ^1^H NMR did not show new visible peaks. However, in the case of L44EDA_0.6, shifts belonging to the phenyl ring and sugar residue were disturbed and new shifts were observed. This could be due to oxidation of the lactose (L) part of L44EDA to lactobionic acid as the possible oxidation product. In contrast to this, neither dimethyl amine nor acetate shifts resulted in any alteration. Therefore, based on the data (given in Supplementary information, Figures S6 and S7) it is reasonable to assume that a larger fraction of the sugar ligands interacts with the formed Au NPs for capping and stabilization (Figure 10). Based on the NMR results, we can conclude that sugar ligands were used in the synthesis to decorate the surface of these metallic NPs, which makes it possible to use a selection of sugar ligands for the design of the surface chemistry of the resultant metallic NPs. The interaction must be strong enough, since heating up to 100 °C did not alter the SPR absorption of Au NPs. According to ^1^H NMR data (given in Supplementary information, Figure S7), interactions of dimethyl amine and acetate with Au^3+^ were short-term, where sugar ligands displaced them and reduced the Au^3+^ that joined the formed Au NPs (Figure 11). However, both dimethyl amine and acetate interacted with the formed Au NP surface. A detailed study to reveal the exact mechanism has been undertaken, and the results will be reported elsewhere.

Protein corona formation is among the key criteria affecting the applicability of nanomaterials in biological applications. Simply, protein corona refers to tagging of proteins on metallic nanostructures that influence the nanomaterials’ fate in biological systems [25]. As an illustrative study, selected Au and Ag NPs were tested for stability against protein corona formation, ionic strength, potassium permanganate, and penicillamine (results are shown in Supplementary information, Figures S8 and S9). The results revealed that both Au and Ag NPs resisted protein corona formation while potassium permanganate triggered re-formation of the NPs (based on SPR peaks) as expected [26]; the absorption peak belonging to KMnO_4_ disappeared within 6 h of incubation due to its consumption. High concentrations of NaCl decreased the intensity of the SPR peak, which is ascribed to the precipitation of Au and Ag NPs (even though it was minimal) with increased ionic strength, thus reducing the intensity of plasmon absorption. Thiol groups in penicillamine can bind to both Au and Ag NPs, so minimal aggregation was observed as expected.

## 4. Conclusions

In conclusion, we demonstrated the synthesis of shape- and size-directed noble metal nanostructures, exemplified by Au and Ag, by using derivatized sugar ligands only. Our results reveal the significant effect of functional groups on the sugar ligands on the morphology, crystallinity, surface chemistry, and extent of agglomeration of the formed nanostructures. For instance, keeping the imine group the same and changing the sugar ligand from CB to L, the morphology of the formed Ag NPs changed from well dispersed cubic (CB3APimine) to rough and aggregated (L3APimine). The sugar ligand/metal ion ratio was shown to be effective in controlling the extent of agglomeration of the formed NPs. This can be exemplified by the CBpAB-Au system, where the decrease of this ratio from 38 to 5 showed a strong agglomeration of the formed spherical Au NPs. In all cases, we could observe the plasmon absorption peaks from the UV-Vis spectra, revealing the low dimensionality and size, where the extent of agglomeration was observed from the plasmon absorption at longer wavelengths for the synthesized NP colloids. An overview of the side group chemistry can be understood by the L ligands effect on the formation of Ag NPs; L3AP and L44EDA yielded smooth, spherical, well-dispersed Ag NPs, while L44ODA yielded smooth, spherical, aggregated NP, and L3APimine, rough, spherical aggregated (>100 nm) NPs. A mixture of amine and imine (L44EDA + L44EDA amine) resulted in an anisotropic particle with mixed morphology and wide range of sizes. Amino salicylic acid functionalization (L5AS) produced sheet-like Ag nanostructures. The use of dimethyl amine and acetate as directing agents also resulted in novel morphologies and various sizes. As examples, L5AS, G5AS, and S5AS ligands formed spherical or sheet-like structures when used alone, which transformed into multicore and rough NPs, revealing the significant effect of these additives on the NPs’ morphology. NPs’ features were shown to be tunable by manipulating the sugar ligand selection and sugar ligand/metal ion ratio. Selected samples were tested for their stability against protein corona formation and ionic strength, where a high chemical stability and resistance to protein coating were observed. The findings show a promising, benign approach for the synthesis of shape- and size-directed Au and Ag nanostructures that can be tuned with the selection, or design, of the chemistry of carbohydrate-derivatives, enabling new applications with direct termination of desired functionalities by using the one-pot synthetic protocols presented here.

## Figures and Tables

**Table 1:** Sugar ligand/metal salt ratio used in the synthesis of Au and Ag nanostructures. Details of molecular structures and abbreviations of the sugar ligands are given in Figure 1.

Sugar Ligand-Metal NP	Sugar Ligand/Metal Salt Mole Ratio					
	Sample Name					
CBpAB-Au	38	26		13		5
	CBpAB-Au_38	CBpAB-Au_26		CBpAB-Au_13		CBpAB-Au_5
CB3Cl-Au	53		8			
	CB3Cl-Au_53		CB3Cl-Au_8a		CB3Cl-Au_8b	
L44ODA-Ag	4					
	L44ODA-Ag					
L44EDA-Ag L44EDA-Au	0.6/61		0.5		2	
	L44EDA-Au_0.6y		L44EDA-Ag_0.5		L44EDA-Ag_2	
L44EDA:L44EDAamine-Ag	2.5/7.4					
	L44EDA:L44EDAamine-Ag*x*					
L3APimin-Ag	1.3					
	L3APimine-Ag					
L3AP-Ag	1.3					
	L3AP-Ag					
CB-3APimine-Ag	0.8					
	CB3APimine-Ag					
G5AS-Ag G5AS-Au	62		62/38^y^/9		1.4/1.7^y^/0.7	
	G5AS-Au_62		G5AS-Au_62*yz*		G5AS-Ag*yz*	
S5AS-Au	102/98^y^/25		41/40^y^/9		62/38^y^/9	
	S5AS-Au_102*yz*		S5AS-Au_41*yz*		S5AS-Au_62*yz*	
L5AS-Au	62/38^y^/9			7/3^y^/2		
	L5AS-Au_62*yz*			L5AS-Au_7*yz*		

*x*: L44EDA:L44EDAimine ratio; *y*: dimethylamine: metal salt mole ratio; *z*: acetate: metal salt mole ratio.

**Table 2:** Various NP morphologies and size distributions of Ag and Au NPs synthesized using derivatized sugar ligands. Details of molecular structures and abbreviation of the sugar ligands are given in Figure 1.

Sugar Ligand	NP Type	NPs’ Morphology; Surface Features and Dispersion Quality	NPs’ Size
CBpAB	Au	Spherical NPs; smooth; well-dispersed	5–40 nm
CB3Cl	Au	Spherical NPs; rough; aggregated	>50 nm (polydisperse)
CB3APimine	Ag	Cubic NPs; well-dispersed	100 nm
L3AP	Ag	Spherical NPs; smooth; well-dispersed	10 nm
L3APimine	Ag	Spherical NPs; rough: aggregated	>100 nm
L44ODA	Ag	Spherical NPs; smooth; well-dispersed	10–30 nm NPs; aggregates >150 nm
L44EDA	Ag	Spherical NPs; smooth; well-dispersed	10–30 nm
L44EDA:L44EDAamine	Ag	Mixture of spherical and non-spherical NPs; slightly aggregated	polydisperse; 100 nm
L5AS	Ag	Sheet-like NPs; well-dispersed	10 nm (thickness)
S5AS	Ag	spherical NPs; smooth; well-dispersed	10 nm
G5AS	Ag	sheet like NPs; irregular; well-dispersed	<30 nm
L5AS	Au	sheet like NPs; irregular; well-dispersed	10 nm (thickness)
**With addition of dimethyl amine and acetate**			
S5AS	Au	Spherical NPs - rough and dendritic; well-dispersed	5–50 nm
G5AS	Au	Rough, multicore NPs; well-dispersed	20–50 nm
L5AS	Au	Rough, multicore NPs; well-dispersed	20–50 nm
L44EDA	Au	Giant truncated crystals; star-shaped NPs; well-dispersed	>5 μm; <100 nm

## Supplementary Materials

The following are available online at https://www.mdpi.com/2079-4991/10/7/1417/s1, Figure S1. UV-Vis absorption spectra of different sugar ligands in water; Table S1: Physicochemical Characteristics and theoretical calculations of the sugar ligands used for the synthesis of nanostructures; Figure S2: (a) TEM image and (b) UV-vis spectrum of Au NPs synthesized from the mixture of cellobiose 4,4′-Diaminodiphenyl sulfone/HAuCl_4_ mole ratio 12; (c) TEM image and (d) UV-vis spectrum of AuNPs synthesized from the mixture of Xylose 4,4’-ethylenedianiline/HAuCl_4_ mole ratio 25; Figure S3: TEM image of AuNPs synthesized from the mixture of Cellobiose 5-aminosalicylic acid/HAuCl_4_ ratio 20; Figure S4: (a) TEM micrograph and (b) UV-vis spectrum of AuNPs synthesized from the mixture of Lactose p-aminobenzoic acid/dimethyl amine/acetate/HAuCl4 ratio 60:1:35:10; Figure S5: SEM micrographs of Ag NPs directly grown on plastic (a,c) and glass (b,d) substrates using the sugar ligands (a,b) L44EDA (c,d) L3AP; Figure S6: ^1^H NMR spectrum of (a) L44EDA_0.6 NPs, and (b) L44EDA ligand (samples dissolved in D_2_O); Figure S7: ^1^H NMR spectrum of LpAB ligand (blue), and LpAB-Au NPs (samples dissolved in D_2_O); Figure S8: Stability tests for L3AP-Ag NPs against protein (P) corona formation, ionic strength (NaCl), potassium permanganate (KMnO_4_) and penicillamine (PA); Figure S9: Stability tests for CBpAB-Au NPs against protein (P) corona formation, ionic strength (NaCl), potassium permanganate (KMnO_4_), and penicillamine (PA).
