# Supplementary material for: On the Effect of Modified Carbohydrates on the Size and Shape of Gold and Silver Nanostructures
Source: Nanomaterials (Basel). 2020 Jul 21;10(7):1417. doi: 10.3390/nano10071417 (PMC7407669; doi:10.3390/nano10071417)
Supplement: Supplementary file 1 [file nanomaterials-10-01417-s001.pdf]

nanomaterials

Supplementary Material

On the Effect of Modified Carbohydrates on the Size and Shape of Gold and Silver Nanostructures

Idris Yazgan[1*], Abdurrahman Gümüş[2], Kutalmış Gökkuş[3], Mehmet Ali Demir[1], Senanur Evecen[4], Hamide Ayçin Sönmez[4], Roland M. Miller[5], Fatma Bakar[4], Ayhan Oral[6], Sergei Popov[7], Muhammet S. Toprak[7*]

[1] Center of Biosensors and Materials, Department of Biology, Faculty of Science and Arts, Kastamonu University, 37150 Kastamonu, Turkey; 115411013@ogr.kastamonu.edu.tr
[2] Department of Electrical and Electronics Engineering, İzmir Institute of Technology, 35430 İzmir, Turkey; abdurrahmangumus@iyte.edu.tr
[3] Department of Environmental Engineering, Kastamonu University, 37150 Kastamonu, Turkey; kgokkus@kastamonu.edu.tr
[4] Center of Science and Art, 37150 Kastamonu, Turkey; senanurevecen@gmail.com (S.E.); sonmezaycn55@gmail.com (H.A.S.); fbakar37@hotmail.com (F.B.)
[5] Department of Chemistry, SUNY Binghamton University, 13902 Binghamton, NY, USA; rmiller8@binghamton.edu
[6] Department of Chemistry, Çanakkale Onsekiz Mart University, 17100 Çanakkale, Turkey; ayhanoral@comu.edu.tr
[7] Department of Applied Physics, KTH Royal Institute of Technology, SE106 91 Stockholm, Sweden
* Correspondence: iyazgan@kastamonu.edu.tr (I.Y.); toprak@kth.se (M.S.T.)

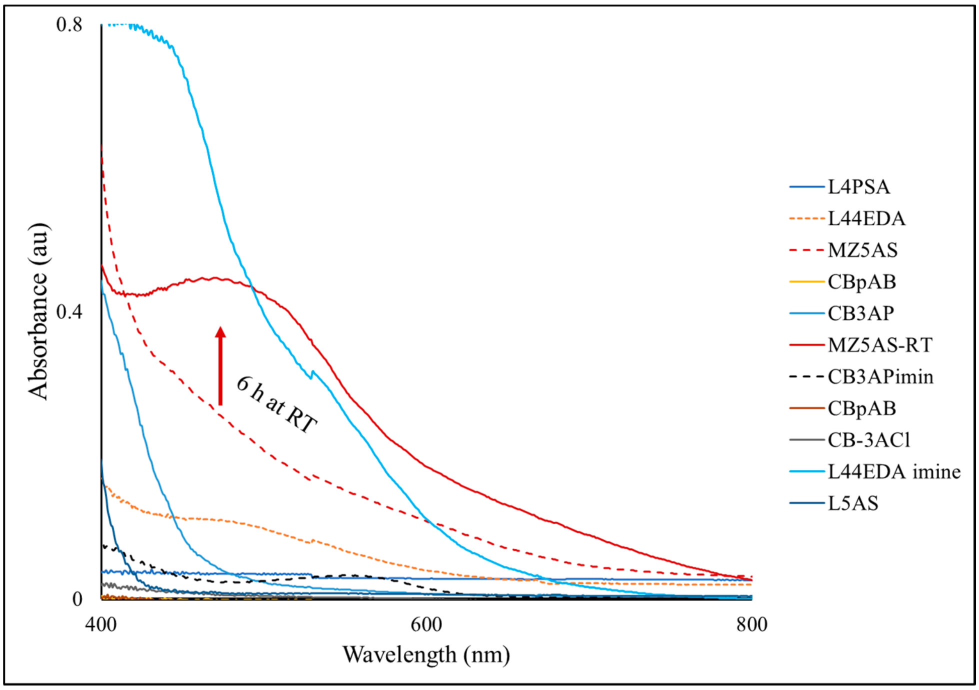

Figure S1. UV-Vis absorption spectra of different sugar ligands in water.

5-amino salicylic acid (5AS) containing sugar ligands, when freshly prepared, did not give any absorbance peak (MZ5AS, Figure S1), however, upon storing at room temperature for 6 h it can give absorbance at ~490 nm. Besides 5AS containing sugar ligands, imine form of the ligands show absorption between 480 and 550 nm (L44EDA imine, Figure S1), which is related to delocalization of π electrons through –C–C– of benzene ring and C=N of imine group. However, pAB containing

ligands (CBpAB, Figure S1) did not provide any absorbance peak upon incubation at room temperature, up to about 60 °C.

Table S1. Physicochemical characteristics and theoretical calculations of the sugar ligands used for the synthesis of the nanostructures. (swissadme.ch was used to calculate the values, and further details if need can be obtained through swissadme.ch [1–3]).

Molecule	Formula	MW (Da)	#Heavy atoms	#Aromatic heavy atoms	Fraction Csp3	#Rotatable bonds	#H-bond acceptors
Cellobiose-4-aminobenzoic acid	$C_{19}H_{29}NO_{12}$	463.43	32	6	0.63	11	12
Cellobiose-3-chloroaniline	$C_{18}H_{28}ClNO_{10}$	453.87	30	6	0.67	10	10
Lactose-4,4'-oxydianiline	$C_{36}H_{56}N_2O_{21}$	852.83	59	12	0.67	22	21
Lactose-4,4'-oxydianiline (single lactose)	$C_{24}H_{34}N_2O_{11}$	526.53	37	12	0.5	12	11
Lactose-4,4'ethylenedianiline	$C_{38}H_{60}N_2O_{20}$	864.88	60	12	0.68	23	20
Lactose-5-aminophenol imin	$C_{18}H_{27}NO_{11}$	433.41	30	6	0.61	9	12
Lactose-5-aminosalicylic acid	$C_{19}H_{29}NO_{13}$	479.43	33	6	0.63	11	13
Lactose-5-aminophenol	$C_{18}H_{29}NO_{11}$	435.42	30	6	0.67	10	11
Galactose-5-aminosalicylic acid	$C_{13}H_{19}NO_8$	317.29	22	6	0.46	8	8
Sucrose-5-aminosalicylic acid	$C_{19}H_{29}NO_{13}$	479.43	33	6	0.63	11	13

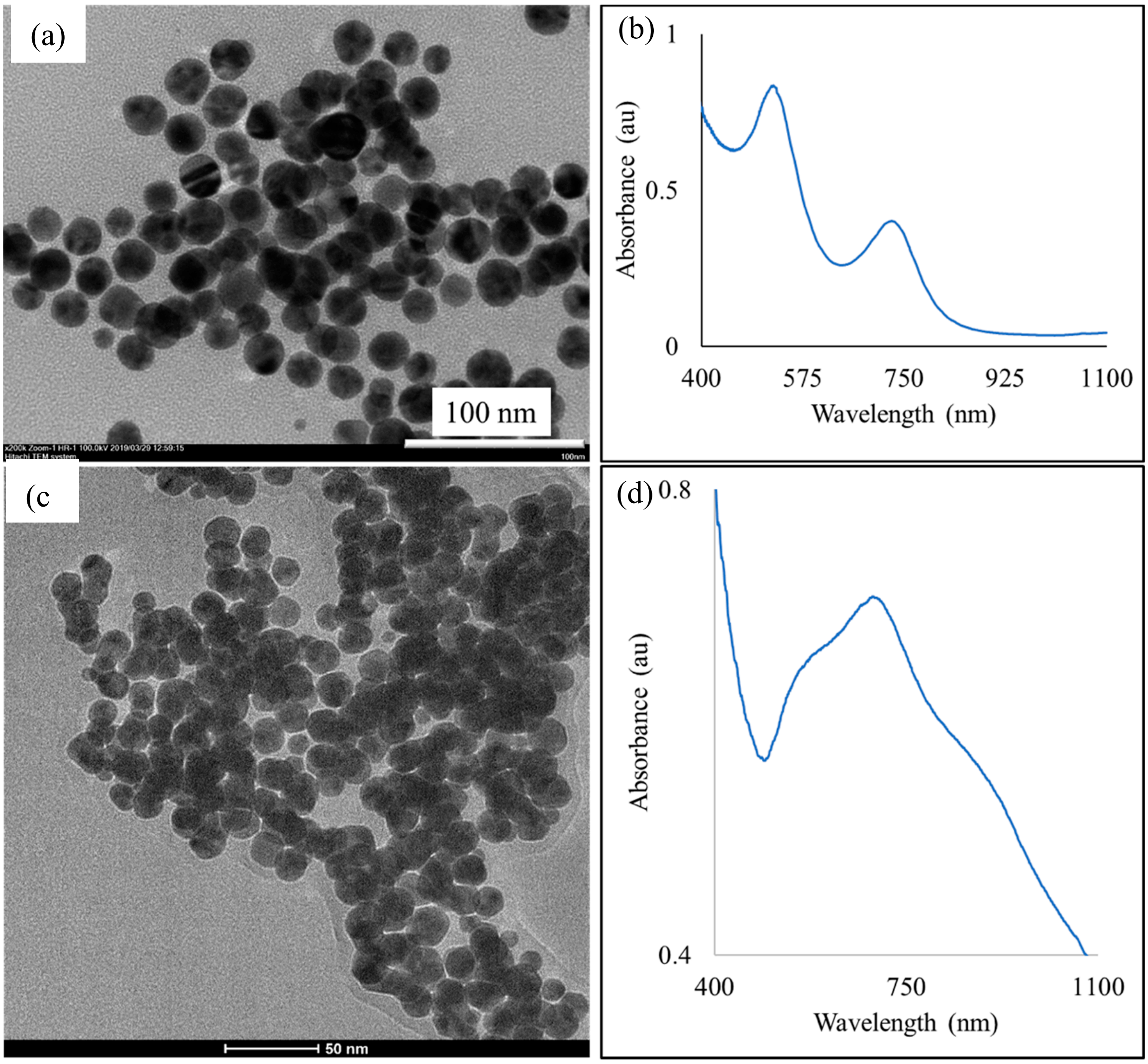

Figure S2. (**a**) TEM image and (**b**) UV-vis spectrum of Au NPs synthesized from the mixture of cellobiose 4,4′-Diaminodiphenyl sulfone/$HAuCl_4$ mole ratio 12; (**c**) TEM image and (**d**) UV-vis spectrum of Au NPs synthesized from the mixture of Xylose 4,4′-ethylenedianiline/ $HAuCl_4$ at mole ratio 25.

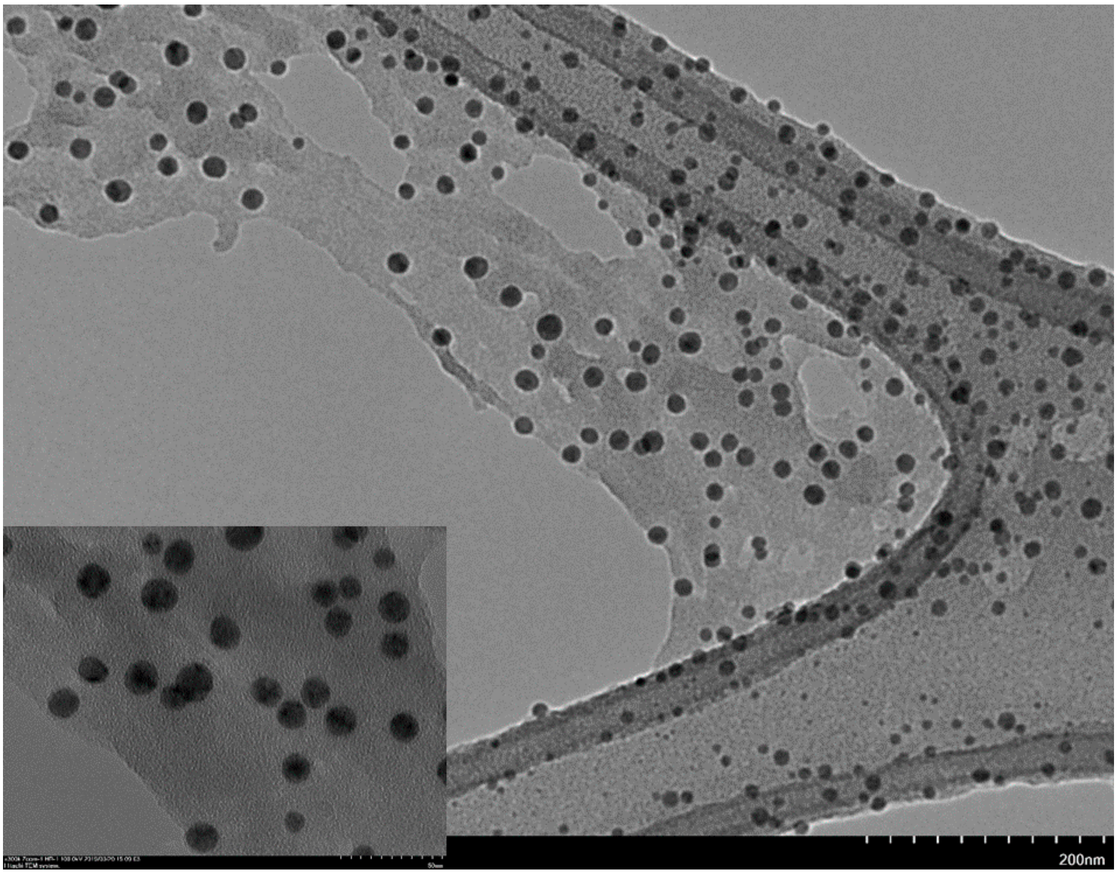

Figure S3. TEM image of AuNP synthesized from the mixture of Cellobiose 5-aminosalicylic acid (CB5AS)/$HAuCl_4$ ratio 20.

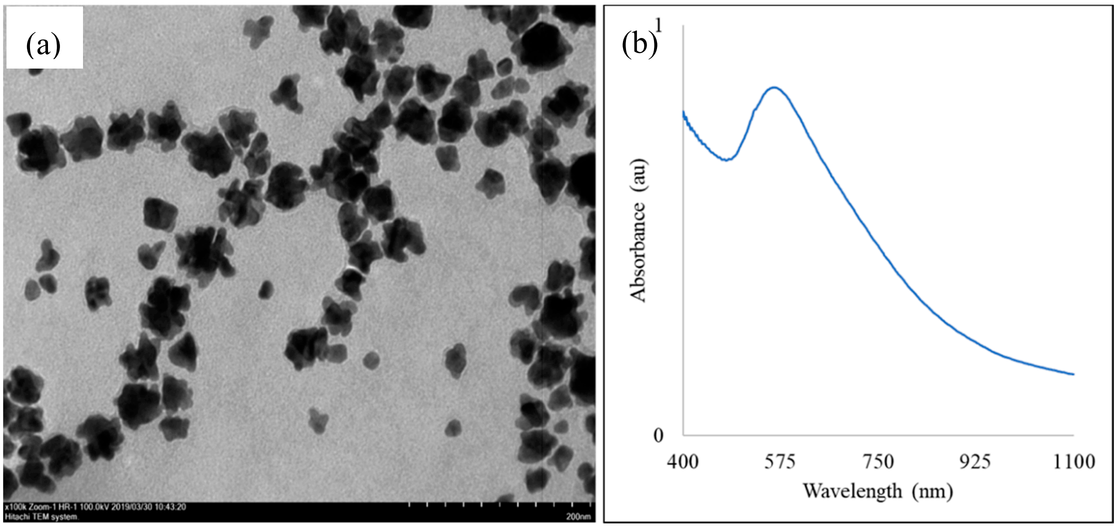

Figure S4. (**a**) TEM micrograph and (**b**) UV-vis spectrum of Au NPs synthesized from the mixture of Lactose p-aminobenzoic acid (LpAB)/dimethyl amine/acetate/HAuCl4 ratio 60:1:35:10.

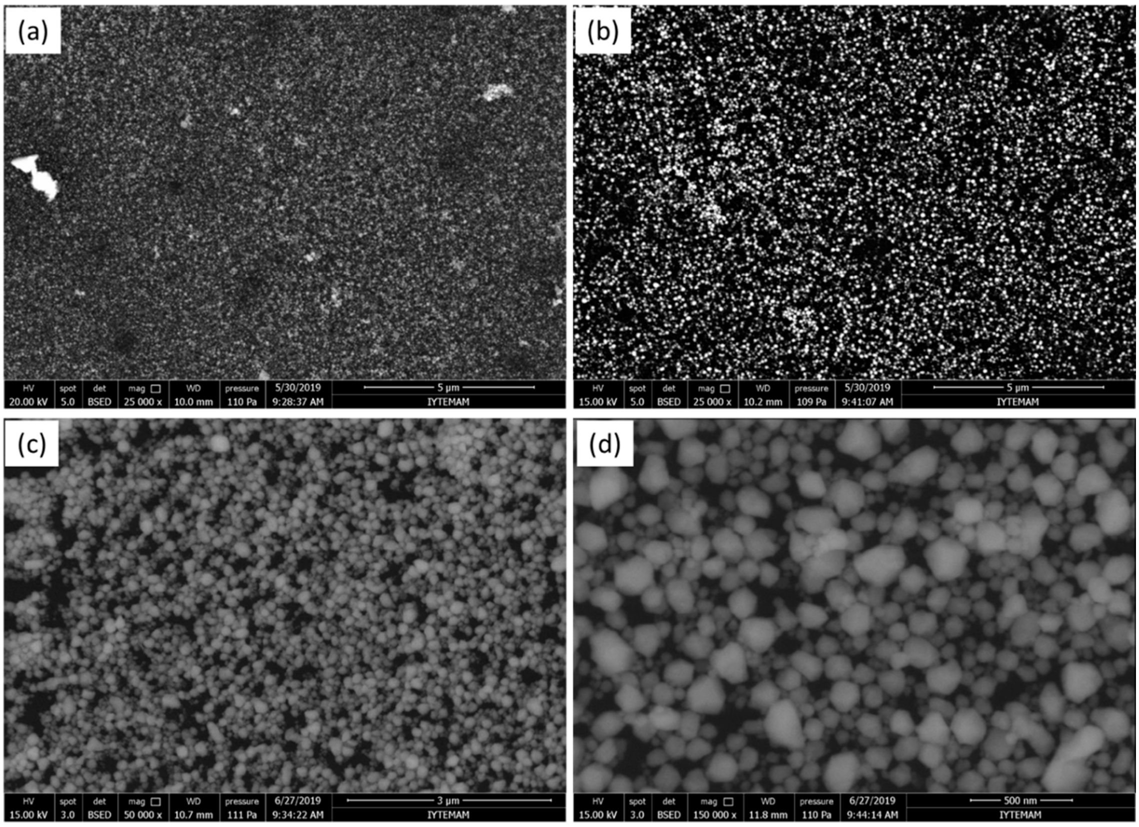

Figure S5. SEM micrographs of Ag NPs directly grown on plastic (**a**,**c**) and glass (**b**,**d**) substrates using the sugar ligands as (a,b) L44EDA, and (c,d) L3AP.

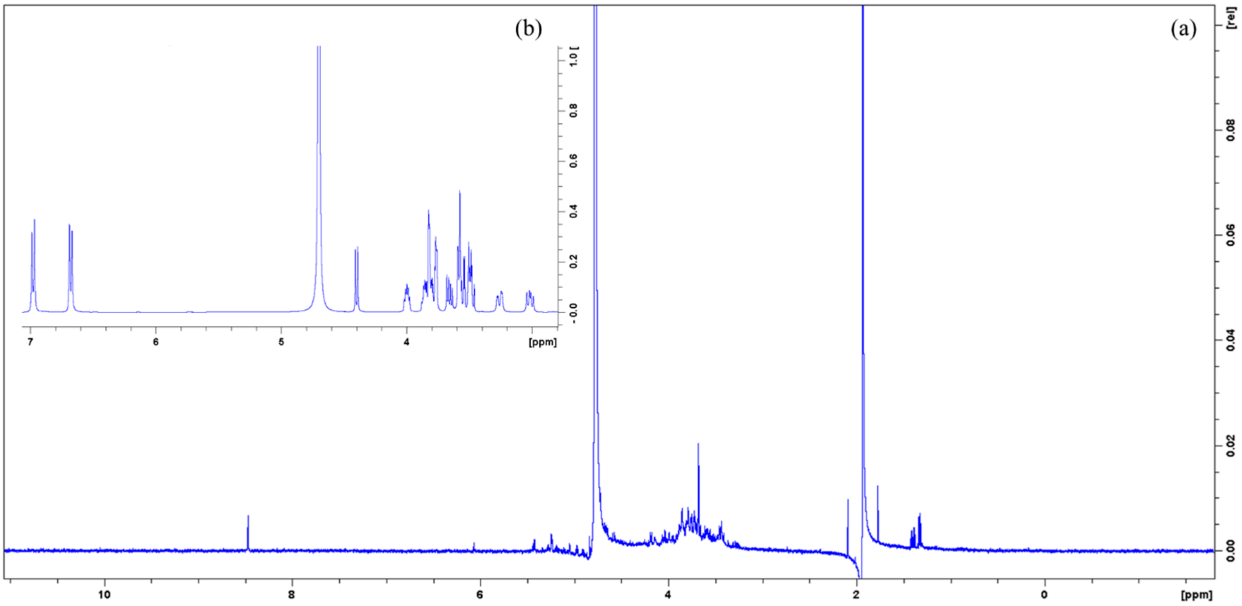

Figure S6. ^{1}H NMR spectrum of (**a**) L44EDA_0.6 NPs, and (**b**) L44EDA ligand (Samples dissolved in D_2O, JEOL NMR-400 MHz).

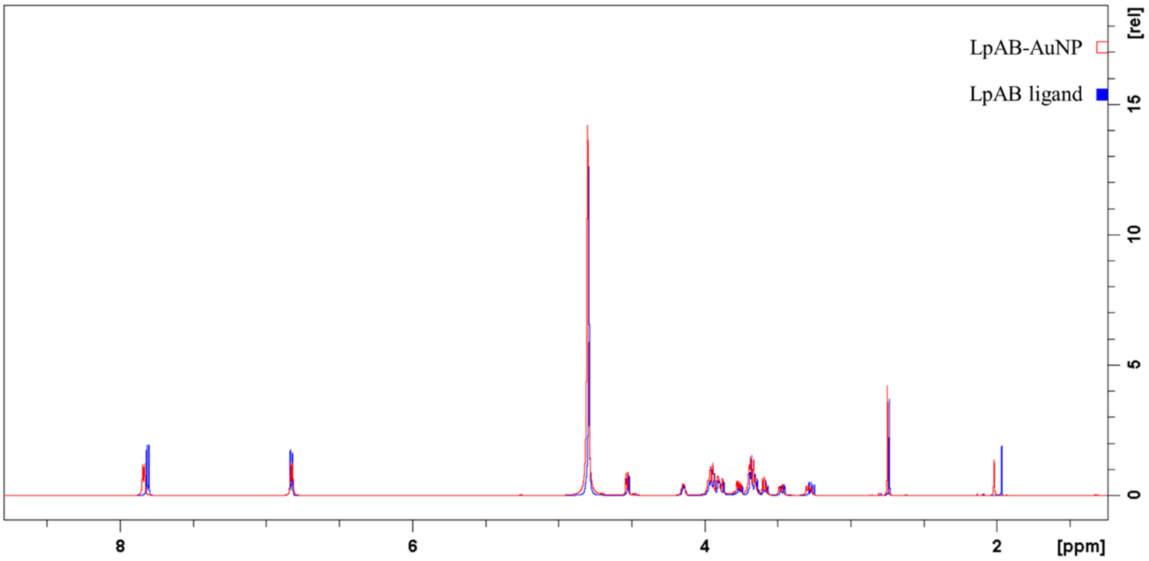

Figure S7. ^{1}H NMR spectrum of LpAB ligand (**blue**), and LpAB-Au NP (**blank**, samples dissolved in D_2O, JEOL NMR-400 MHz).

Stability of AuNPs and AgNPs

The stability of synthesized NP systems is essential for their successful use. Therefore, we studied the protein adsorption, effect of ionic strength and oxidizing agent represented by potassium permanganate on selected Ag and Au NP systems. Main effects/causes for the tests are briefly described below.

Protein corona formation: Protein corona formation is among the key players affecting the applicability of nanomaterials in biological applications. Simply, protein corona refers to tagging of proteins on metallic nanostructures that influence nanomaterials' fate in biological systems [4].

Thiol group susceptibility test: Penicillamine (3,3-Dimethyl-L-cysteine) was used as model thiol group containing molecule because of its good solubility in water and structural similarity to cysteine. Stability of ligand shell of NPs is essential in their applications [5]. Free thiol groups can displace the ligands on the gold [6] and silver NPs [7].

Effect of Ionic Strength: Ionic strength can pose threat to the stability of NPs as reported in literature [8]. Hereby we tested up to 1.0 M NaCl concentration on both Au and Ag nanostructures.

Effect of Potassium Permanganate: Potassium permanganate is able to ionize Ag NPs [9] and Au NPs [10], which may cause dissolution and re-formation of NPs.

Results of these tests performed on L3AP-Ag NP system are presented in Figure 11a, where the plasmon signal of Ag NPs was monitored to observe any change or aggregation. The SPR absorption observed for Ag NPs is centered around 410 nm, which did not change its position significantly.

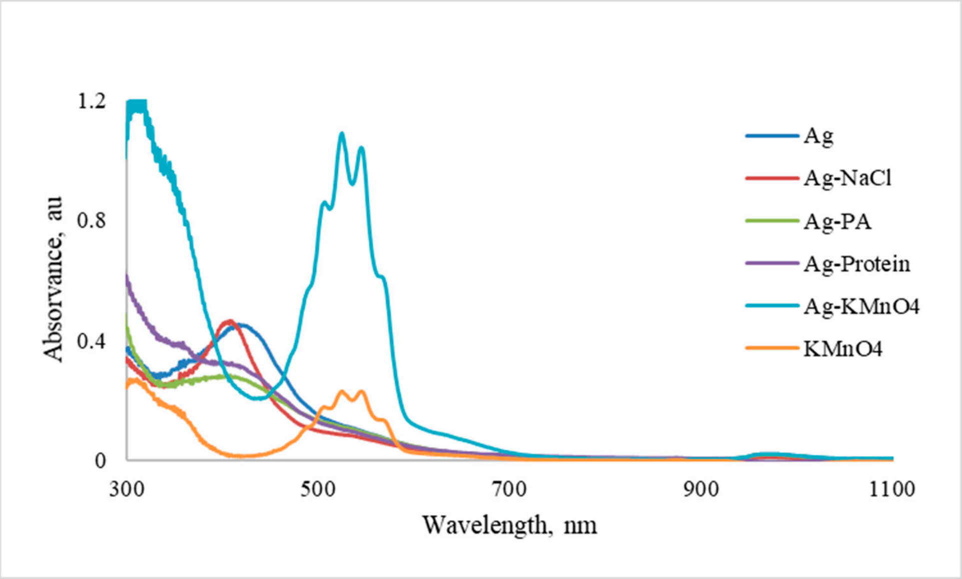

Figure S8. Stability tests for L3AP-Ag NPs against protein (P) corona formation, ionic strength (NaCl), potassium permanganate ($KMnO_4$) and penicillamine (PA).

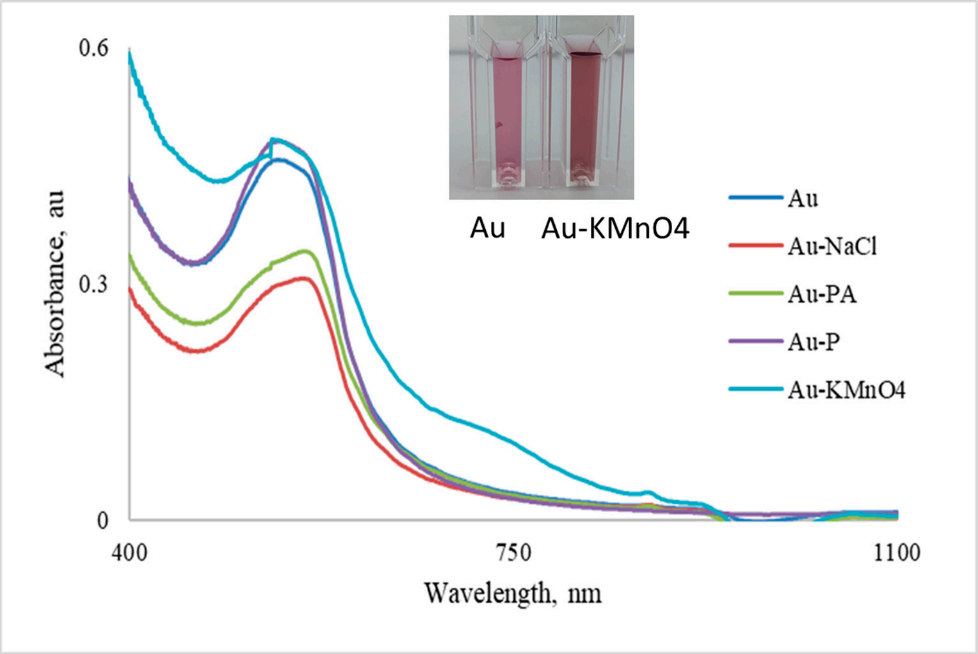

Figure S9. Stability tests for CBpAB-Au NPs against protein (P) corona formation, ionic strength (NaCl), potassium permanganate ($KMnO_4$) and penicillamine (PA).

References

1. Daina, A.; Zoete, V. A BOILED-Egg To Predict Gastrointestinal Absorption and Brain Penetration of Small Molecules. *ChemMedChem* **2016**, 1117–1121. https://doi.org/10.1002/cmdc.201600182.
2. Daina, A.; Michielin, O.; Zoete, V. ILOGP: A simple, robust, and efficient description of n-octanol/water partition coefficient for drug design using the GB/SA approach. *J. Chem. Inf. Model.* **2014**, *54*, 3284–3301. https://doi.org/10.1021/ci500467k.
3. Daina, A.; Michielin, O.; Zoete, V. SwissADME: A free web tool to evaluate pharmacokinetics, drug-likeness and medicinal chemistry friendliness of small molecules. *Sci. Rep.* **2017**, *7*, 42717. https://doi.org/10.1038/srep42717.
4. Charbgoo, F.; Nejabat, M.; Abnous, K.; Soltani, F.; Mohammad, S.; Alibolandi, M.; Shier, W. T.; Steele, T. W. J. Gold nanoparticle should understand protein corona for being a clinical nanomaterial. *J. Control. Release* **2018**, *272*, 39–53. https://doi.org/10.1016/j.jconrel.2018.01.002.
5. Woehrle, G. H.; Brown, L. O.; Hutchison, J. E. Thiol-functionalized, 1.5-nm gold nanoparticles through ligand exchange reactions: Scope and mechanism of ligand exchange. *J. Am. Chem. Soc.* **2005**, *127* (14), 2172–2183.
6. Stobiecka, M.; Deeb, J.; Hepel, M. Ligand exchange effects in gold nanoparticle assembly induced by oxidative stress biomarkers: Homocysteine and cysteine. *Biophys. Chem.* **2010**, *146*, 98–107. https://doi.org/10.1016/j.bpc.2009.11.001.
7. López-Lorente, Á. I.; Soriano, M. L.; Valcárcel, M. Analysis of citrate-capped gold and silver nanoparticles by thiol ligand exchange capillary electrophoresis. *Microchim. Acta* **2014**, *181*, 1789–1796. https://doi.org/10.1007/s00604-014-1218-5.
8. Řezanka, P.; Řezanková, H.; Matějka, P.; Král, V. The chemometric analysis of UV-visible spectra as a new approach to the study of the NaCl influence on aggregation of cysteine-capped gold nanoparticles. *Colloids Surfaces A Physicochem. Eng. Asp.* **2010**, *364*, 94–98. https://doi.org/10.1016/j.colsurfa.2010.05.001.
9. Qin, J.; Sui, M.; Yuan, B.; Wang, J.; Yuan, Z.; Xu, G. The decay of silver nanoparticles in preoxidation process. *Sci. Total Environ.* **2018**, *619–620*, 1618–1627. https://doi.org/10.1016/j.scitotenv.2017.10.140.
10. Calderón-jiménez, B.; Johnson, M. E.; Bustos, A. R. M.; Murphy, K. E.; Winchester, M. R.; Baudrit, J.R.V. Silver Nanoparticles®: Technological Advances , Societal Impacts , and Metrological Challenges. *Front. Chem.* **2017**, *5*, 1–26. https://doi.org/10.3389/fchem.2017.00006.
